# CD8+ T Cell Subsets as Biomarkers for Predicting Checkpoint Therapy Outcomes in Cancer Immunotherapy

**DOI:** 10.3390/biomedicines13040930

**Published:** 2025-04-09

**Authors:** Rosaely Casalegno Garduño, Alf Spitschak, Tim Pannek, Brigitte M. Pützer

**Affiliations:** 1 Institute of Experimental Gene Therapy and Cancer Research, Rostock University Medical Center, 18057 Rostock, Germany; rosaely.casalegno@med.uni-rostock.de (R.C.G.); alf.spitschak@med.uni-rostock.de (A.S.); tim.pannek@med.uni-rostock.de (T.P.); 2 Department Life, Light & Matter, University of Rostock, 18059 Rostock, Germany

**Keywords:** cancer immunotherapy, CD8+ T cell subsets, biomarker, ICB, TILs, melanoma, NSCLC

## Abstract

The advent of immune checkpoint blockade (ICB) has transformed cancer immunotherapy, enabling remarkable long-term outcomes and improved survival, particularly with ICB combination treatments. However, clinical benefits remain confined to a subset of patients, and life-threatening immune-related adverse effects pose a significant challenge. This limited efficacy is attributed to cancer heterogeneity, which is mediated by ligand–receptor interactions, exosomes, secreted factors, and key transcription factors. Oncogenic regulators like E2F1 and MYC drive metastatic tumor environments and intertwine with immunoregulatory pathways, impairing T cell function and reducing immunotherapy effectiveness. To address these challenges, FDA-approved biomarkers, such as tumor mutational burden (TMB) and programmed cell death-ligand 1 (PD-L1) expression, help to identify patients most likely to benefit from ICB. Yet, current biomarkers have limitations, making treatment decisions difficult. Recently, T cells—the primary target of ICB—have emerged as promising biomarkers. This review explores the relationship between cancer drivers and immune response, and emphasizes the role of CD8+ T cells in predicting and monitoring ICB efficacy. Tumor-infiltrating CD8+ T cells correlate with positive clinical outcomes in many cancers, yet obtaining tumor tissue remains complex, limiting its practical use. Conversely, circulating T cell subsets are more accessible and have shown promise as predictive biomarkers. Specifically, memory and progenitor exhausted T cells are associated with favorable immunotherapy responses, while terminally exhausted T cells negatively correlate with ICB efficacy. Ultimately, combining biomarkers enhances predictive accuracy, as demonstrated by integrating TMB/PD-L1 expression with CD8+ T cell frequency. Computational models incorporating cancer and immune signatures could further refine patient stratification, advancing personalized immunotherapy.

## 1. Immune Checkpoint Blockade—Still an Open Road in Cancer Therapy

Immunotherapies represent a milestone in the treatment of cancer. These are therapeutic approaches that stimulate or suppress the immune system in order to combat tumor growth and metastasis, achieving a survival improvement for patients [1,2]. Immuno-based treatments of cancer comprise strategies such as (i) adoptive cell transfer of specific T cells, including chimeric antigen receptor (CAR) T cells; (ii) cancer vaccines designed to boost the immune system’s response to tumors; and (iii) immune checkpoint blockade (ICB) [2,3]. The latter has attracted considerable attention in recent years due to its impressive, long-lasting results [2,4]. ICB therapies aim to achieve a self-sustaining cancer immunity cycle with minimal autoimmunity [5]. The principle is based on humanized monoclonal antibodies targeting immune checkpoint proteins, such as programmed cell death protein 1 (PD1), its ligand (PD-L1), and cytotoxic T lymphocyte-associated protein 4 (CTLA-4) [6]. Particularly, patients with metastatic melanoma, non-small cell lung carcinoma (NSCLC), and head and neck cancers have benefited from ICBs [7].

To date, the Food and Drug Administration (FDA) has approved several PD1 inhibitors (nivolumab, pembrolizumab, cemiplimab and dostarlimab), PD-L1 inhibitors (atezolimumab, avelumab and durvalumab), a lymphocyte-activation gene 3 (LAG-3) inhibitor (relatlimab), and CTLA-4 inhibitors (ipilimumab, tremelimumab) [1,6,8]. Here, PD1/PD-L1 blockade is currently the main ICB treatment for various types of cancer, such as melanoma, renal cell carcinoma (RCC), head and neck squamous cell carcinoma (HNSCC), and gastroesophageal adenocarcinoma (GEA) [1]. Despite the promising results that have been achieved, the clinical benefit is still limited, as evidenced by the number of partial responders and non-responders, and the prevalence of side effects [2,6,7]. For instance, treatment with pembrolizumab or nivolumab has an objective response rate (ORR) of 40-45% in patients with melanoma [1]. In addition, immune-related adverse events (irAEs) remain a major concern in the treatment of patients [6,8,9]. In a retrospective study conducted by Wang et al. (2024), the occurrence of irAEs was observed in 42% of cancer patients undergoing therapy with different ICB agents [10], while in another report, up to 96% of treated patients experienced irAEs [11]. The most frequently occurring irAEs are dermatologic toxicities, colitis, pneumonitis, hepatitis and endocrine and cardiac toxicities [5,10]. As a consequence, patients with irAEs require therapy interruption or additional systemic treatment to manage the undesirable side effects, which ultimately leads to a lower overall survival (OS) rate compared to patients without irAEs [10,12]. To limit systemic toxicity, new approaches are being pursued, including simultaneously engaging and blocking inhibitory receptors on a given cell, such as bispecific antibody-like molecules that bind and block concomitantly or independently to both PD1 and LAG-3 [13] or PD1 and CTLA-4 [14,15]. Although these engineered molecules lead to certain anti-tumor activity, a significant number of patients do not respond to the treatment and/or experience irAEs [13,16].

Currently, it is becoming increasingly challenging to improve the efficacy of clinically established therapies. Given the limited response rates, treatment-related toxicities, and the fact that irAEs can be associated with mortality and significant lifelong morbidity, there is a pressing need for predictors and novel strategies that enable efficient and prescient management of both cancer and co-occurring immune-related diseases [3,12]. This will ultimately translate into improved and personalized cancer immunotherapy for patients.

In this review, we summarize the current results of ICB therapy in different cancers and its impact on circulating and tumor-resident CD8+ T cell subsets, along with current limitations and challenges in stratifying patients by current biomarkers. Finally, we highlight potential solutions and outline future perspectives in treatment and forecasting of patients.

## 2. Molecular and Cellular Drivers of Cancer Progression as Modulators of the Immune Response

During tumor development, neoplastic cells acquire intrinsic properties through genetic and epigenetic alterations, deregulated signaling, and metabolic changes, while manipulating immune cells to create an immunosuppressive tumor microenvironment (TME). Various immune cells, including T and B lymphocytes, macrophages, dendritic cells, natural killer (NK) cells, and neutrophils, exhibit high plasticity and can either promote or inhibit metastasis. Cancer cells evade the immune response by inducing immune cell exhaustion, recruiting immunosuppressive cells, and producing inhibitory cytokines and immune checkpoint molecules. This dynamic interplay between cancer and immune cells facilitates escape from the immune system, metastasis, and influences the efficacy of immunotherapy. Several transcription factors, ligand–receptor interactions, exosomes, and secreted factors are pivotal drivers of cancer progression and are synergistically intertwined with immunoregulatory pathways and functions [12].

In particular, cancer stem cells (CSC), which have the ability to self-renew and differentiate, exhibit remarkable immune resistance, a critical feature that was overlooked by most immunotherapies. This omission has led to frequent treatment failures, often resulting in cancer relapse [17]. Their biological activities are regulated by distinct pluripotent factors, including OCT4, Sox2, Nanog, KLF4, and MYC. Additionally, multiple intracellular signaling pathways—such as Wnt, NF-κB, Notch, Hedgehog, JAK-STAT, PI3K/AKT/mTOR, TGF/SMAD and PPAR—as well as extracellular factors, such as vascular niches, hypoxia, tumor-associated macrophages (TAMs), cancer-associated fibroblasts (CAFs), mesenchymal stem cells, extracellular matrix and exosomes, play a crucial role in regulating CSC development [18]. Transcriptional dysregulation of the MYC oncogene is one of the most frequent events in aggressive human cancers, but it is also integral to the regulation of the tumor immune microenvironment. It specifically promotes the expression of immunosuppressive factors and blocks regulators of immune activation. Yang and colleagues revealed that Myc inhibition can shift the functions of CD8+ T cells by disturbing the homeostasis of regulatory CD4+ T (Treg) cells and triggering the differentiation of resting Treg (rTreg) cells into activated Tregs (aTreg). Depending on the Myc level, certain T cell subsets have conferred differential sensitivity to the pharmacological inhibition of Myc [19,20]. In addition, Wang et al. (2018) demonstrated that the function of Tregs within tumor infiltrates is dependent on the Enhancer of Zeste Homolog 2 (EZH2) protein. Blockade of EZH2 caused Tregs to obtain pro-inflammatory activity, thereby enhancing cancer immunity [21]. A critical tumor-intrinsic axis of pharmacological interest for cancer therapy is the vascular endothelial growth factor (VEGF)/VEGF receptor (VEGFR) system. Inhibition of this pro-angiogenic pathway by VEGFR2 kinase blockade has been shown to be effective in various cancers [22], as it reduces immunosuppression by preventing the recruitment of immature dendritic cells (DCs), myeloid-derived suppressor cells (MDSCs), and Tregs [23,24]. Anti-angiogenic agents targeting this axis promote a pro-inflammatory microenvironment [25].

Another well-known transcription factor that has been identified in recent years as a major mediator of cancer cell aggressiveness is E2F1. While it contributes to apoptosis in response to DNA damage as part of a tumor surveillance mechanism, abundant expression of E2F1 in advanced cancer cells drives metastatic transformation by promoting epithelial-to-mesenchymal transition (EMT), neoangiogenesis, extravasation, and genomic instability, which correlates with poor prognosis [26,27,28]. Network modeling and gene expression profiling have identified tumor-specific receptor signatures in which high E2F1, TGFBR1, and FGFR1 activity fosters invasive phenotypes [29]. The oncogenic activity of E2F1 depends to a great extent on coregulators that form protein–protein interaction complexes, which enhance metastasis-related gene expression. Thus, the E2F1-MTA1 complex promotes lung metastasis by upregulating the production of HAS2 and hyaluronic acid (HA), which in turn recruits TAMs of type M2 phenotype. Disruption of this complex impairs the formation of a metastatic TME by reducing HAS2/HA levels [30]. Based on clinical data from TCGA, we recently demonstrated that increased levels of E2F1, STAT3, and IL6, impacting cancer stemness and progression, correlate with CD4+ T helper type 2 (Th2) infiltration, while blocking Th1 in primary and metastatic melanoma. Conversely, factor depletion shifted this Th1/Th2 imbalance in the TME toward Th1 antitumor immunity [12]. We also observed significant deregulation of the transcriptional landscape and cytokine secretion of CD8+ T cells depending on tumor-intrinsic E2F1 levels. This includes a marked increase in IL6 release under coculture conditions. Since the IL6-STAT3 signaling pathway inhibits the differentiation of cytotoxic CD8+ T cells, which limits anti-PD-L1 activity during ICB treatment, these findings underscore the detrimental impact of high-E2F1 on CD8+ T cells and its ability to promote therapy resistance.

**Figure 1:**
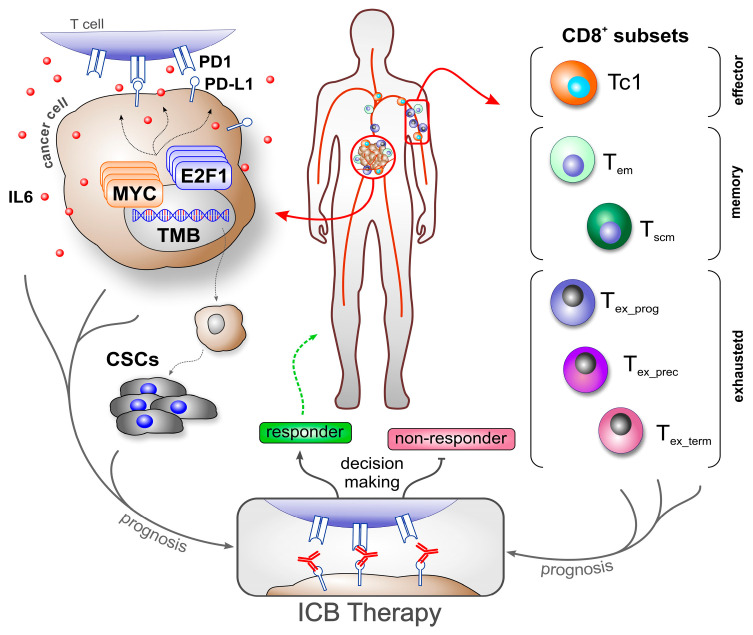
Overview of cancer- and immune cell-related biomarker use to predict ICB therapy outcomes. Molecular and cellular factors, such as transcription factors (e.g., MYC, E2F1), cytokine release (e.g., IL6), checkpoint molecule expression (e.g., PD-L1), and cancer stem cells, drive cancer progression and contribute to an immunosuppressive tumor microenvironment, making them valuable biomarker candidates (left). When combined with specific CD8^+^ T cell subsets, these biomarkers enable more precise prognosis of immune responses, supporting patient stratification and informed decision making for the application and development of effective immunotherapy strategies. ICB: immune checkpoint blockade; TMB: tumor mutational burden; T_em_: effector memory T cell; T_scm_: T memory stem cell; T_ex_prog_: progenitor exhausted T cell; T_ex_prec_: precursor exhausted T cell; T_ex_term_: terminally exhausted T cell.

A significant challenge in ICB therapy is the lack of reliable biomarkers for predicting treatment response [1,31,32,33]. This is also reflected in the current use of FDA-approved biomarkers on cancer cells, such as tumor mutational burden (TMB), mismatch repair deficiency/microsatellite instability-high (MSI-H), and PD-L1 expression. Especially, PD-L1 expression in immune or cancer cells is one of the most commonly used biomarkers for ICB today. However, individual biomarkers cannot accurately predict the outcome in all patients [34,35]. PD-L1 predicted the clinical outcome of only 28.9% of cancer patients treated with ICBs [36] and showed no correlation to treatment outcome determination in patients with melanoma, Merkel cell carcinoma, RCC, or hepatocellular carcinoma [6,36]. PD-L1 in combination with TMB enabled more accurate predictions of certain cancers. For example, Ricciuti et al. (2022) found a positive association with outcomes in 57% of patients with NSCLC under PD1/PD-L1 treatment who had both high TMB and high PD-L1 [37]. In contrast, the same biomarker combination was not able to predict outcomes in a cohort study of patients with GEA [38]. These discrepancies might be attributed to variations in the expression levels depending on tumor stage (primary versus metastatic), tumor region, and even different phases of cancer progression and treatment [6,38]. External factors such as smoking play an additional role in the expression of PD-L1 [39]. Consequently, relying on PD-L1 expression alone remains insufficient for accurate decision making [34]. As E2F1 and MYC are associated with resistance to anti-PD1 therapy [40,41], and given the crucial role of CSCs, integrating these parameters into biomarker-based therapeutic strategies could further improve predictability and treatment success (Figure 1).

Ultimately, as cancer-killing CD8+ T cells are the main targets of ICB, measuring their population, status, and functionality to predict their response to immunotherapy has received great attention in recent years.

## 3. CD8+ T Cell Heterogeneity in Cancer—Using Population Subsets as Biomarkers

Most mature T cells express either CD4 or CD8 co-stimulatory molecules, which has classified them into two populations, CD4+ and CD8+, respectively. Antigen-presenting cells (APCs) located in lymph nodes present antigens to CD8+ T cells in the context of major histocompatibility complex class I (MHC-I). The T cells recognize the presented antigens via their T cell receptor (TCR). Antigen-specific clones are then activated and differentiated and they proliferate into distinct subsets. The polarization of the T cell subsets is determined by the time/strength of TCR: MHC-I binding (signal 1), costimulatory molecules (e.g., CD28:CD80/CD86, signal 2), and cytokines (signal 3); this results in either cytotoxic T lymphocytes (CTLs, more recently referred to as Tc1), Tc2, Tc9, Tc17, Tc22, or Tregs [42,43,44]. Tc1 cells are highly efficient in killing virus-infected and malignant cells and are the primary targets of immunotherapy. A high abundancy of Tc1 cells in cancer patients is associated with improved survival. Understandably, tumors with a high Tc1 infiltration rate show a better response to ICB [43]. An overview of CD8+ T cell subsets used as biomarkers is shown in Figure 1, and their clinical association is summarized in Table 1.

### 3.1. Pan CD8+ T Cells

A high infiltration of immune cells in tumors (“hot tumors”) is typically a good prognostic factor that correlates positively with effective ICB treatment [45]. Similarly, the density of tumor-infiltrating lymphocytes (TILs), particularly tumor-associated antigen (TAA)-specific CD8+ T cells, has been linked to favorable clinical outcomes in numerous cancers [46,47], and is therefore considered a potential predictive biomarker [6,48]. Indeed, frequencies of CD8+ T subsets in tumor biopsies are strong indicators for the clinical benefit of ICB [6,9,49,50,51,52,53,54,55]. Meta-analyses performed by Geng et al. (2015) [50] and Li et al. (2021) [52] found a strong positive correlation between CD8+ TILs and good outcomes in cancer patients treated with conventional checkpoint inhibitors (i.e., CTLA-4, PD1, and PDL1). Furthermore, in order to overcome resistance to current ICB agents, attempts have recently been made to block additional inhibitory receptors, including LAG-3 in combination with PD1 [54,56,57,58,59,60], TIGIT in combination with PD-(L)1 +/− chemotherapy [55,61,62,63,64], and TIM3 in combination with PD-(L)1 or LAG-3 +/− chemotherapy [64,65]. Most of these studies are still in ongoing clinical trials. LAG-3 inhibits anti-tumor Tc1 activity and contributes to their exhaustion. *LAG-3* is highly expressed in tumor-infiltrating CD8+ T cells in various cancers, but particularly high in samples from melanoma tumors [66]. Thereafter, beneficial effects on CD8+ TIL responses have been reported in advanced melanoma and in patients with HNSCC treated with combined anti LAG-3 and PD1 [54,59,66]. Immune infiltration following treatment was associated with a better OS [54], which could be explained by the increased IFNγ response and improved TCR signaling despite maintaining an exhaustion phenotype [54,59,60]. The synergistic, non-redundant effects of blocking both LAG-3 and PD1 have also been further described in mouse models of cancer, where deletion of these inhibitory receptors in CD8+ T cells resulted in enhanced effector function [60]. Interestingly, increased expression of the inhibitory heterodimer receptors NKG2A+ and CD94+ on CD8+ T cells, both in murine cancer models and in patients with advanced melanoma, resulted from blocking both LAG-3 and PD1 [60], possibly as a compensatory mechanism to prevent immunopathology. Of note, blocking of NKG2A using monoclonal antibodies (monalizumab) boosts both NK cell and CD8^+^ T cell responses [67]. Although PD1- and LAG-3-deficient CD8+ TILs showed increased proliferation, cleaved caspase-3 was also increased, suggesting increased cell death [60]. Additionally, combinational therapy blocking both TIGIT and PD-L1 showed slightly better OS in patients with NSCLC compared to a control group receiving anti-PD-L1 plus placebo; CD8+ effector TILs were associated with improved ORR in patients receiving both ICB agents [55].

Taking tumor biopsies can be challenging in some cases, and patient follow-up is not a viable option with this approach. Consequently, noninvasive blood-based biomarkers represent an excellent way to both predict patient outcomes/responses to immune checkpoint inhibitors and monitor treatment progress over time [48]. This approach is based on the assumption that specific clones located within the tumor can also be found in the peripheral blood of cancer patients [53]. Additionally, peripheral blood mononuclear cells (PBMCs) collected at baseline have shown that patients who respond well to ICB therapy have a pre-existing immune profile [33]. Therefore, the measurement of circulating CD8+ T cell populations could be an indicator of antitumor/therapy response, and the circulating cell signature prior to therapy could classify patients into responders and non-responders. Indeed, both high and increased frequencies of circulating total CD8+ and proliferative Ki67+ CD8+ T cells have been associated with clinically good outcomes in patients receiving ICB therapies [54,55,66,68]. Notably, Ki67+ CD8+ T cells are found in the tumor center of responders to ICB [49].

Furthermore, T cells that differ in the expression of costimulatory molecules such as CD28 could give an indication of patient response. CD28 provides signals that are indispensable for T cell activation and survival. Hence, the CD28+ CD8+ subset has been identified as a major player in the response to checkpoint inhibitor administration [69]. In a retrospective study, patients who responded to therapy which blocks the PD1/PD-L1 axis displayed higher frequencies of circulating CD28+ CD8+ cells, and their progression free survival (PFS) and OS were longer. An excessive proportion of this subset was also associated with severe irAEs [69]. Therefore, CD28+ CD8+ T cells might not only be a predictive marker for ICB therapies but also for treatment-related toxicity. In contrast to this report, another publication observed no correlation between clinical outcome and circulating CD28+ CD8+ cells in patients with cancer treated with anti-PD1/PD-L1 [33].

The migration of T cells towards tumor lesions is of great importance for an adequate antitumor immune response. As activated lymphocytes upregulate the expression of chemokine receptors [70], their increased expression, such as of the CX3C motif chemokine receptor 1 (CX3CR1), have been used as biomarkers to predict the success of ongoing immunotherapeutic approaches [71,72]. As early as four weeks after the initiation of combined chemo/anti-PD1 therapy, an increase in circulating CX3CR1+ CD8+ T cells of at least 10 percent correlates with clinical benefit and positive ORR in patients with advanced NSCLC, and ultimately with improved survival [72]. Similar results were obtained in patients with NSCLC receiving anti-PD1 monotherapy [71].

### 3.2. Memory CD8+ T Cells

Following the eradication of cancer cells, the majority of T cell clones die during the contraction phase. However, some clones survive and constitute the memory compartment (Figure 1), which undergoes immediate proliferation upon antigen re-stimulation and plays a pivotal role in cancer control [43,73]. The identification of CD8+ memory subsets is based on surface molecules, with central memory expressing CD45RO^hi^ CD28^hi^ CCR7^hi^ CD62L^hi^ CD45RA^lo^, effector memory (Tem) expressing CD45RO^hi^ CD28^hi^ CCR7^lo^ CD62L^lo^ CD45RA^lo^, terminal effector expressing CD45RA^hi^ CD28^lo^ CCR7^lo^ CD62L^lo^ CD45RO^lo^, memory stem cell (Tscm) expressing CD45RA^hi^ CCR7^hi^ CD95^hi^ CD45RO^lo^, and tissue-resident memory (Trm) expressing CD103^hi^ CD69^hi^ CD45RO^hi^ CCR7^lo^ CD62L^lo^ CD45RA^lo^ [74,75]. Subsequently, researchers have examined these populations and their effect on clinical responses before and after ICB therapies. Particularly, CD8+ Tem cells have been associated with a good prognosis in cancer patients [68,76,77,78]. Baseline circulating CD8+ Tem frequencies correlate with better OS and clinical response in patients with advanced melanoma receiving anti-CTLA-4 [68,77,78], as well as in other cancer patients treated with PD1/PD-L1 axis inhibitor [33]. Also, patients with metastatic melanoma responding to CTLA-4 inhibition but not to anti-PD1 therapy had a higher ratio of circulating CCR7- CD45RO+ CD8+ Tem cells compared to baseline (≥30% of total circulating CD8+ cells) [76]. Moreover, patients with NSCLC who responded better to anti PD1/PD-L1 therapy had higher counts of peripheral CD8+ Tscm before treatment [79]. Furthermore, the CD103+ CD8+ Trm subset in the tumor was associated with the anticancer response in patients with HNSCC and triple-negative breast cancer (TNBC) to ICB in combination with chemotherapy [80]. Likewise, Edwards and colleagues (2018) measured the frequency of CD103+ CD8+ TILs before and after anti-PD1 treatment in patients with metastatic melanoma and found that this subgroup expanded following ICB therapy in a very small cohort of responder patients [81]. Further studies with larger patient samples are proposed to confirm these results. Combination ICB therapy enhances the proliferation of memory subsets, as observed in patients with HNSCC receiving PD1 and LAG-3/CTLA-4 blockade. Anti-PD1 combined with anti-CTLA-4 expands Tem and Trm CD8+ TILs in responders, whereas anti-PD1 combined with anti-LAG-3 reactivates and reprograms exhausted CD8+ TILs into Tem and Trm [59].

### 3.3. Exhausted CD8+ T Cells

When prolonged antigen stimulation occurs, as is the case in the TME, CD8+ T cells do not undergo the typical memory pathway but become exhausted. T cell exhaustion occurs upon chronic antigen/TCR stimulation in combination with several factors such as inflammatory cytokines, hypoxia, and glucose deprivation, all exhibited in the hostile tumor microenvironment [82,83]. Exhausted T cells show reduced proliferation and cytotoxic function and fail to control tumor progression. Advances in single-cell RNA sequencing (scRNA-seq) reveal the great heterogeneity of exhausted T cells, including precursor, progenitor, intermediate/transitory, and terminal [9,42,82,83]. Subsets of exhausted T cells are classified based on the progressive expression of inhibitory receptors like CTLA-4, PD1, T cell immunoglobulin and mucin domain-containing protein 3 (TIM3), LAG-3, P-selectin glycoprotein ligand-1 (PSGL-1), CD39, CD73, and hematopoietic progenitor kinase 1 (HPK1) [32,42,78,82,83,84]. ICB attempts to reactivate exhausted anti-tumor T cells by blocking the signaling pathways that contribute to the maintenance of the exhaustion state. Therefore, researchers have focused on the response of the exhausted subsets to therapy and are investigating them as biomarkers in both tumor samples and peripheral blood. It is worth noting that progenitor exhausted T cells are long-lived, self-renewing cells that can differentiate into terminally exhausted subsets and proliferate in response to PD1 blockade [5,9,83,85]. Thereafter, self-renewing stem-like exhausted cells, precursor, and progenitor subsets have been positively correlated with ICB therapy. Liu et al. (2022) observed an increment in precursor exhausted T cells, defined as CXCL13+ TIM3- CD8+, in tumor samples from patients with NSCLC who responded to anti-PD1 treatment, in contrast to tumors that did not respond to PD1 treatment and had an accumulated proportion of terminally exhausted CD8+ T cells [53]. Similar results examining an increase in a progenitor exhausted PD1+ TCF1+ CD8+ population were reported by Miller and co-authors (2019) in patients with melanoma receiving anti CTLA-4 combined with anti-PD1 therapy. Of note, the authors also reported no significant differences in the CD8+ T cell subset analyzed between pre-treatment biopsies of responders and non-responders in the same patient cohort [9]. TCF1, encoded by *TCF7*, is a transcription factor that supports differentiation, self-renewal, and memory-related features [78]. Remarkably, *TCF7*, as well as other genes related to the activation and survival of CD8+ T cells, were enriched in tumor biopsies obtained from patients with metastatic melanoma responding to ICB treatment (Sade-Feldman et al. 2019). Also, responders to anti-PD1 inhibitors show increased TCF1+ CD8+ TILs, whereas non-responders showed increased TCF1- CD8+ TILs [78]. Moreover, Daud et al. (2016) showed a positive correlation between PD1^hi^ CTLA-4^hi^ CD8+ TILs and clinical benefit in patients with metastatic melanoma subjected to PD1 blockage [86]. Similarly, increased frequencies of PD1+ CD8+ T cells in the TME were associated with better outcomes of PD1 blockade therapies in head and neck cancer [87], NSCLC, and GC [31].

The presence of exhausted subsets has also been tested in liquid biopsies. An increased frequency in circulating CD38+ TIM3+ CD8+ T cells was observed following combined anti-LAG-3 and anti-PD1 therapy in patients with advanced melanoma [54]. Furthermore, this combined therapy not only expanded LAG-3+ CD8+ T cells but also boosted their cytotoxic phenotype in another studied cohort [66]. Patients with cancer that have high frequencies of circulating PD1+ CD8+ cells at baseline correlated with a better outcome of PD1/PD-L1 axis blocking therapy [33]. Additionally, a low baseline frequency of circulating PD1+ CD8+ and NK cells combined with a high expression of soluble PD-L1 (sPD-L1) was negatively associated with the response to PD1/PD-L1 inhibition in patients with advanced NSCLC [88]. Of note, PD1 expression is also associated with the activation of circulating and tumor-resident T cells [12,84]. The extent to which the clustering of CD8+ T cells based on PD1+ expression reflects either exhausted or freshly activated cells remains to be elucidated by the expression of additional markers.

The response to PD1/PD-L1 inhibitor therapy in patients with NSCLC could also be predicted by high proportions of the exhausted subset CD39+ CD8+ TILs [89]. Interestingly, CD39 is also expressed on CD8+ T regs [90,91], which may explain the results obtained by Koh and colleagues (2020). A lower frequency of CD39+ CD8+ at baseline and follow-up was associated with longer survival and beneficial PD1 therapy in patients with advanced NSCLC. The correlation was stronger in addition to a low frequency of monocytic MDSCs [92]. Furthermore, scRNA-seq of CD8+ T cells from metastatic melanoma tumor biopsies of patients responding to ICB treatment revealed low expressions of both *ENTPD1* and *HAVCR2*, genes that encode for CD39 and TIM3, respectively. Murine experiments confirmed this association, showing that CD39- TIM3- CD8+ T cells have antitumor activity [78]. In the same way, a lower frequency of circulating CD73+ PD1+ CD8+ cells has been shown to correlate with better outcomes in patients with advanced melanoma after PD1 inhibitor treatment [32]. CD73 expression on immunoregulatory Tregs has also been reported [93,94]. Although blockade of the checkpoint molecules LAG-3, PD1/PD-L1, and TIGIT aims to activate anti-tumor CD8+ cytotoxic T cells, they also reactivate and expand exhausted Tregs, contributing to therapy resistance [31,55,66,95,96]. Surprisingly, Guan and colleagues (2024) found that combined treatment with anti-TIGIT and anti-PD-L1 increased Tregs and TAMs in tumors, yet correlated with improved ORR and OS in patients with NSCLC [55]. It is important to note that these studies are based on CD4+ Tregs and do not take CD8+ Tregs into account.

On the other hand, terminally exhausted cells are short-lived and unresponsive to ICB [5,83,85]. They show a low expression of the transcription factor TCF1 and high expressions of TOX and Eomes, as well as surface molecules PD1 and TIM3 [85]. Predictively, a high proportion of terminally exhausted CD8+ T cells in blood and within the tumor is reflected in ICB ineffectiveness. Zhang et al. (2024) observed that a high tumor infiltration of HPK1+ PD1+ TIM3+ CD8+ T cells was rather linked to a poor immune response of therapeutic ICB in patients with NSCLC [84]. Also, in patients with advanced NSCLC treated with anti-PD1, a lower percentage of the terminal exhausted peripheral Eomes+ PD1+ CD8+ subgroup showed an improved outcome [97].

### 3.4. T Cell Distribution in the TME

A number of studies also suggest that the location of CD8+ T cells in the TME might be an even better predictor of treatment efficacy, as cell interactions affect cellular responses. Tumeh et al. (2014) reported on the association between CD8+ TILs at the invasive margin of metastatic melanoma tumors and a clinical benefit for PD1 blockade therapy. The proximity between PD1+ and PD-L1+ cells (assuming that they are mainly CD8+ T cells and tumor cells, respectively) also showed good clinical results in the same cohort of patients [49]. Another report demonstrated that a higher number of CD8+ TILs, either intratumoral and/or in the stroma, led to better OS and PFS in patients treated with ICB, with stromal accumulation being a stronger biomarker [52].

### 3.5. Bottlenecks in Biomarker Application—Conflicting Roles of CD8+ T Cells

Notwithstanding the above reports, CD8+ TILs remain inconsistent as predictive biomarkers. Some investigations found a lack of association between circulating and tumor infiltrating CD8+ T cells, and clinical outcomes of ICB [32,88,92,98,99,100]. These discrepancies could be related to several factors, including tumor stage (primary versus metastatic), ICB agent(s), time-dependent tumor sampling associated with ICB treatment, technical variability (i.e., immunohistochemistry, flow cytometry, multiplex imaging, antibodies/clones), markers/CD8+ T cell subset, aggregation (or absence) of lymphocytes in certain parts of the tumor tissue (the so-called tertiary lymphoid structures, TLS), location of CD8+ cells in the TME (intratumoral, stroma, invasive margin), responsiveness of exhausted T cells to therapy, individual/patient-related factors (age, gender, comorbidities, smoking status), or even cancer entities. Li et al. (2021) pointed to a strong correlation between CD8+ TILs from NSCLC and other solid tumors and improved OS with ICB treatment, but not in melanoma [52]. In the same way, the density of CD8+ TILs was not predictive of the therapeutic efficacy of ICB in patients with metastatic TNBC [100]. In addition, Zhang et al. (2024) showed that the effective density of CD8+ cells in tumor samples from patients with NSCLC undergoing anti-PD1/PD-L1 therapy did not correlate with the OS, whereas a more specific HPK1+ PD1+ TIM3+ CD8+ subset indicated a negative correlation [84]. Therefore, the standardization of CD8+ T cell markers should be considered a key factor for a uniform comparison of studies.

Higher expression levels of circulating PD1+ CD8+ T cells at baseline were associated with a worse outcome in advanced NSCLC treated with anti-PD1 [97]. Furthermore, high levels of peripheral CXCR4+ CD8+ T cells in treatment-naive patients with NSCLC correlated with poorer OS after PD1 inhibition [98]. As reported by Nabet and colleagues (2020), fewer baseline circulating CD8+ T cells were associated with the clinical benefit of anti-PD1/PD-L1 therapy in patients with advanced NSCLC. The authors speculated that this was a consequence of increased CD8+ T cells’ homing at tumor sites, although this hypothesis was not tested [99].

**Table 1:** Overview of CD8+ T cell subsets used as biomarkers and their clinical association. adv.: advanced; ChTx: chemotherapy; CITE-seq: cellular indexing of transcriptomes and epitopes by sequencing; CyTOF: cytometry by time of flight; CTLA-4: cytotoxic T lymphocyte-associated protein 4; FC: flow cytometry; FFPE: formalin fixed paraffin embedded; GC: gastric cancer; HNSCC: head and neck squamous cell carcinoma; IHC: immunohistochemistry; irAE: immune-related adverse events; MDSCs: myeloid-derived suppressor cells; met.: metastatic; mIF: multispectral immunofluorescence; NK: natural killer cells; NSCLC: non-small cell lung carcinoma; OS: overall survival; PB: peripheral blood; PBMCs: peripheral blood mononuclear cells; PD1: programmed cell death protein 1; PD-L1: programmed death ligand 1; PET: positron emission tomography; PFS: progression free survival; scRNA-seq: single-cell RNA sequencing; SKCM: skin cutaneous melanoma; TCR-seq: single-cell TCR sequencing; Tem: effector memory T cells; TILs: tumor-infiltrating lymphocytes; TME: tumor microenvironment; TNBC: triple negative breast cancer; Trm: tissue-resident memory T cells; Tscm: T memory stem cell; ^89^Zr: zirconium-89. T tumor tissue; B peripheral blood. * Detected in tumor tissue and secondary lymphoid organs.

Entity	Therapy	Subset	Biomarker	Origin	Technique	Conclusion	Ref.
NSCLC	Anti PD1 and ChTx	Precursor exhausted	CXCL13+ TIM3- CD8+ TILs	T	scTCR-seq	Increased precursor exhausted CD8+ T cells in responsive tumors after treatment.	[53]
adv. SKCM	Anti PD1 and CTLA-4	Progenitor exhausted	PD1+ TCF1+ CD8+ TILs	T	Quantitative multiplex immunofluorescence in pre- and post-treatment biopsies	No significant difference in PD1+ TCF1+ CD8+ T cell frequencies were seen in pre-treatment biopsies of responsive tumors vs. non-responsive. However, increased frequency of the studied subset was significantly associated with clinical outcome.	[9]
met. SKCM	ICB agents		TCF1+ CD8+ TILs		scRNA-seq and IHC of tumor samples	TCF1+ CD8+ TILs predict response to therapy and were correlated to a positive outcome in patients. TCF7+ CD8+ T cells were enriched in tumor biopsies obtained from metastatic SKCM patients responding to ICB treatment.	[78]
NSCLC	Anti PD1/PD-L1	Terminally exhausted	HPK1+ PD1+ TIM3+ CD8+ TILs	T	FFPE-stained tissue by multiplex immunofluorescence	High infiltration of HPK1+ PD1+ TIM3+ CD8+ TILs correlated to poor prognosis in patients receiving ICB.	[84]
adv. NSCLC	Anti PD1		Eomes+ PD1+ CD8+ T cells	B	FC of PB at baseline and during treatment	Low percentage of circulating Eomes+ PD1+ CD8+ associated with an improved outcome. Higher levels of CD8+ T cells correlated with longer OS and PFS. No correlation was found in patient survival and CD8+ ratio relative to a specific CD4+ Treg subset.	[97]
NSCLC	Anti PD1/PD-L1	Exhausted	CD39+ CD8+ TILs	T	Multiplex IHC or immunofluorescence	Higher proportion of CD39+ CD8+ TILs found in responders to therapy.	[89]
adv. NSCLC			PD1+ CD8+ T cells	B	FC of PB at baseline	Low frequencies of baseline PD-1+ CD8+ and NK cells combined with high plasma sPD-L1 was negatively associated with therapy response.	[88]
adv. NSCLC	Anti PD1		CD39+ CD8+ T cells		FC of PB at baseline and follow-ups	Lower frequencies of CD39+ CD8+ T cells associated with better OS. Lower frequencies of both circulating CD39+ CD8+ T cells and monocytic MDSCs showed a stronger correlation with OS.	[92]
NSCLC and GC			PD1+ CD8+ TILs	T	FC, CyTOF	Increased frequencies of PD1+ CD8+ T cells in the TME were associated with better outcomes.	[31]
adv. SKCM			CD73+ PD1+ CD8+; PD1+ CD8+ T	B	FC at baseline before treatment	Low frequency of circulating CD73+ PD1+ CD8+ and PD1+ CD8+ at baseline associated with clinical benefit of therapy.	[32]
met. SKCM			PD-1^hi^ CTLA-4^hi^ CD8+ TILs	T	FC of tumor samples pre- and post-treatment	Increased frequencies of PD1^hi^ CTLA-4^hi^ CD8 TILs strongly correlated with response to therapy.	[86]
adv. NSCLC	Anti PD1/PD-L1		PD1+ CD8+ T cells	B	FC of PB at baseline	Low frequencies of baseline PD-1+ CD8+ and NK cells combined with high plasma sPD-L1 were negatively associated with therapy response.	[88]
various					FC of PBMCs at baseline, and week 6 and 20 post-treatment	High frequencies of circulating PD1+ CD8+ at baseline correlated to a better outcome.	[33]
adv. SKCM	Anti PD1 and LAG3		CD38+ TIM3+ CD8+ T cells	B	FC at baseline and 4 weeks after treatment	Increased frequency of CD38+ TIM3+ CD8+ T cells following treatment.	[54]
met. SKCM	Anti PD1 and LAG3 (+/−prior anti PD1/CTLA-4)		LAG3+ CD8+ T cells	B	scRNA, TCR-seq, FC in pre-treatment, and 4 and 12 weeks PB after therapy	Increased frequency of LAG3+ CD8+ cells in responders after treatment.	[66]
NSCLC and GC	Anti PD1	Exhausted vs. immunosuppressive	PD1 expression in CD8+ and CD4+ Tregs	T	FC, CyTOF	Higher PD1 expression in CD8+ T cells and lower expression of PD1 in CD4+ Tregs correlated to a favorable antitumor response.	[31]
met. SKCM	Anti CTLA-4	Tem	CCR7- CD45RO+ CD8+ T cells	B	FC of PBMCs pre- and post-treatment	SKCM patients responding to CTLA-4 blocking therapy had a higher ratio of CCR7- CD45RO+ CD8+ memory cells compared to baseline.	[76]
adv. SKCM			CD27+ CD28 + CD8+ T cells		FC of PBMCs before treatment and follow-ups	High effector memory CD8+ T cell frequencies at baseline correlated with good clinical outcome.	[68]
			CD45RA- CCR7- CD8+ T cells		FC of PB before, during, and at the end of treatment	Increased CD8+ Tem cell frequencies at the end of the treatment correlated with better OS and clinical response.	[77]
various	Anti PD1/PD-L1				FC of PBMCs at baseline, and week 6 and 20 post-treatment	Baseline CD8+ Tem cell frequencies correlate with better OS and clinical response.	[33]
adv. NSCLC		Tscm	CD45RA+ CD95+ CD62L+ CD45RO- CD8+ T cells	B	FC of PB	Therapy responders had higher counts of CD8+ Tscm prior to therapy.	[79]
met. SKCM	Anti PD1	Trm	CD103+ CD8+ TILs	T	FC and quantitative multiplex immunofluorescence on treatment-naive and undergoing tumor samples	CD103+ CD8+ T cells in the TME expanded after therapy. Patients showed improved survival.	[81]
adv. HNSCC	Anti PD1 and ChTx				scRNA-seq, FC, multiplex immunofluorescence of FFPE tumor samples before treatment	Increased CD103+ CD8+ TIL density in patients responding to therapy.	[80]
adv. NSCLC	Anti PD1	Migratory	CXCR4+ CD8+ T cells	B	FC of PBMCs before therapy	High frequencies of peripheral CXCR4+ CD8+ T cells in treatment-naive patients correlated to worse OS.	[98]
	Anti PD1 and ChTx		CX3CR1+ CD8+ T cells		FC of PBMCs at baseline and follow-ups	CX3CR1+ CD8+ T cells correlate with clinical benefit.	[72]
NSCLC	Anti PD1					At least 20% increase in circulating CX3CR1+ CD8+ T cells correlated to clinical benefit. This subset can be used as an early on-treatment biomarker.	[71]
various	Anti PD1/PD-L1	CD28+ CD8+	CD28+ CD8+ T cells	B	FC of PB at baseline before Tx	Higher frequencies of circulating CD28+ CD8+ T cells were associated with responsive patients who received blocking of the PD1/PDL1 pathway. Excessive accounts of the measured subset are indicative of severe irAEs.	[69]
					FC of PBMCs at baseline, and week 6 and 20 post-treatment	No correlation between clinical outcome and circulating CD28+ CD8 T cells.	[33]
met. SKCM	Anti PD1	Pan CD8	CD8+ TILs	T	Quantitative IHC, quantitative multiplex immunofluorescence, and NGS for TCR performed pre- and during treatment tumor samples	Reduction in tumor correlates to proliferation of CD8+ TILs. Association of CD8+ TILs at the invasive margin of met. SKCM tumors and clinical benefit. Less diverse TCR repertoire associated with a better outcome.	[49]
NSCLC	Anti PD1 and ChTx				scRNA-seq, IHC	Higher frequencies of CD8+ T cells in responsive tumors.	[53]
adv. TNBC	Anti PD-L1 and ChTx				IHC of serial tumor biopsies	CD8+ TILs were not predictive of the therapeutic efficiency.	[100]
various	ICB agents				Meta-analysis	Higher accounts of CD8+ T cells in either intratumor and/or stroma showed a better OS and PFS in ICB-treated patients; however, stromal was a stronger biomarker.	[52]
met. SKCM	Anti CTLA-4		CD8+ T cell ratio in TME		IHC of serial tumor biopsies	CD8+ TIL density was higher in early on-treatment tumors from responders vs. non-responders.	[51]
	Anti PD1 (+/− prior CTLA-4)					Patients responsive to therapy a had higher CD8+ T cell ratio at the tumor core relative to the invasive margin in early on-treatment biopsies.	
NSCLC	Anti PD1/PD-L1		CD8+ T cells	B	FC and RNA-seq of PB at baseline	Fewer circulating CD8+ T cells were associated with successful therapy.	[99]
adv. SKMC	Anti PD1 and LAG3		CD8+ T cells	B/T	scRNA at baseline and 4 and 16 weeks after treatment	Combined ICB therapy improved cytotoxicity and TCR signaling despite persistence of the exhausted phenotype.	[54]
adv. HNSCC	Anti-PD1 +/− LAG-3/CTLA-4		CD8+ TILs	T	scRNA-seq, scTCR-seq, CITE-seq, and mIF of PPFE at baseline and post-treatment	Anti PD1 and LAG-3 therapy reactivates exhausted CD8+ TILs and increases TCR diversity and CD8+ TILs. Anti-PD1 and CTLA-4 therapy does not change the exhausted phenotype, and rather increases Tem and Trm CD8+ TILs.	[59]
NSCLC	Anti TIGIT and PD-L1		CD8+ T cells	B/T	Bulk RNA-seq of pre-treatment tumor samples; scRNA-seq of pre-treatment PBMCs, and 2, 3, and 9 weeks after treatment	Treatment resulted in increased frequency of circulating non-naive CD8+ T cells. Improved OS and PFS after combined ICB therapy associated with CD8+ effector TILs.	[55]
various	Anti PD1/PD-L1 and/or CTLA-4	CD8+ cells (mostly T cells)	^89^Zr-labeled CD8+ cells	T *	PET scans tracking ^89^Zr-labeled CD8+ T cells before and approx. 30 days after treatment. Corroboration by IHC staining of CD8 T cells in tumors before and during treatment	Tracking biodistribution of ^89^Zr-labeled CD8 T cells in cancer patients. Patients with higher tracker uptake had better OS.	[101]
NSCLC	Anti PD1	PD1+ cells (mostly T cells)	^89^Zr-labeled PD1+ cells		PET scans tracking ^89^Zr-labeled PD1-expressing cells at 2, 4 and 7 days post injection.	Uptake of ^89^Zr-labeled PD1 correlated with OS, PFS and response to therapy.	[102]
					PET scans tracking ^89^Zr-labeled PD1-expressing cells	^89^Zr-labeled anti-PD1 uptake correlated to clinical response without statistical significance.	[103]

## 4. Converging Pathways: Novel Roads and Integrative Strategies to Enhance T Cell Response Prediction in ICB Therapy

Given the limitations of a single universal marker in predicting ICB therapy outcomes across all cancer patients [34,35,101], multimodal, multiparametric approaches hold great promise for precision medicine. Integrating CD8+ T cell signatures with additional biomarkers—such as PD-L1 tumor expression, sPD-L1, TMB, myeloid lineage populations, neutrophil-to-lymphocyte ratio, and Th1/Th2 CD4+ T cells—may enhance predictive accuracy. A positive correlation between CD8+ TILs, neoantigen load, and high TMB in cancers such as melanoma, lung, and bladder is associated with better clinical outcomes than low TMB. Conversely, cancers like glioma, breast, and prostate, where this correlation is absent, show poor responses to ICB therapy [104]. Transcriptomic analysis of retrospective data from patients with GC identified an eight-gene CD8+ T cell signature predictive of clinical outcome. This signature includes a cytokine (*TGFB1*), an inhibitory (*PDCD1*), and a chemokine receptor (*CXCR4*) and was used to predict ICB response in GC. Patients with a high-risk CD8-associated gene signature and worse OS had a higher tumor immune dysfunction and exclusion (TIDE) score, along with low inhibitory receptor expression, TMB, and MSI, suggesting limited benefit from ICB therapy. However, their clinical relevance remains unconfirmed [105]. A recently proposed immunoscore, based on CD8 and PD-L1 expression in tumor samples, aims to predict the success of PD-(L)1 blockade therapy. This standardized and reproducible approach evaluates cell clustering, location, density, and T cell proximity to PD-L1+ cells using a univariate Cox model and demonstrated in a validation cohort that a low index of these parameters is a negative predictor of long-term ICB benefit [106]. Overall, integrating multiple biomarkers may improve patient stratification into responders and non-responders.

### 4.1. Determination of T Cell Subset Ratios in Cancer Patients

The ratio of CD8+ T cells in different compartments of the TME has been linked to the stratification of responders from non-responders. It was previously reported that patients with metastatic melanoma responding to anti PD1 had a higher CD8+ T cell ratio in the tumor center compared to the invasive margin in early on-treatment biopsies [51]. Moreover, scRNA-seq of tumor samples showed that a higher proportion of CD8+ cells with increased expressions of memory, activation, and survival-associated genes compared to exhaustion genotype of CD8+ T cells correlated with a positive outcome in patients with metastatic melanoma receiving ICB treatment. Furthermore, a higher number of TCF1+ CD8+ versus TCF1- CD8+ TILs was found in responders, whose survival was longer [78].

The presence of regulatory T cells (formerly called suppressor T cells) in the TME can suppress the favorable anti-tumor Tc1 immune response. However, the study by Kumagai et al. (2020) revealed no correlation between the ratio of CD8+ T cells relative to CD45RA- CD25^hi^ Foxp3^hi^ CD4+ Tregs in the TME and a benefit of anti-PD1 therapy [31]. Ottonello et al. (2020) also observed a lack of correlation between the ratio of circulating CD8+ T cells to CD25+ CD127- CD39+ Foxp3+ CD4+ Tregs and the clinical benefit of ICB [97]. Both studies, however, only considered CD4+ Tregs and not all regulatory T lymphocytes (i.e., CD8+ Tregs).

PD1+ CD8+ T cells are currently controversial as biomarkers, with some studies associating them with a good response to ICB [31,33,87,88], while others do not [32,68,97]. The detection of PD1 expression in CD8+ T cells could have misleading results since they are a very heterogeneous population that also includes CD8+ Tregs [44,107], and anti-PD1/PDL1 therapy activates both PD1+ cytotoxic and immunosuppressive T cells [31,95,96]. Indeed, mouse experiments show that higher PD1 expression in CD8+ T cells and lower expression of PD1 in CD4+ Tregs correlates with a favorable antitumor immune response under anti-PD1 treatment. The same results were predicted and validated in tumor patient samples, but not in blood-circulating T cells [31]. In this context, baseline PD1+ CD8+ relative to PD1+ CD4+T cells is associated with a good clinical outcome in patients who receive PD1/PD-L1 inhibitor [108].

### 4.2. TCR Profiling

Bystander CD8+ T cells account for the majority of T cells surrounding the tumor. They are not TAA-specific T cells but accumulate in the vicinity of cancerous cells without killing the tumor [109]. Therefore, TCR profiling to determine T cell specificity against TAAs has been used as a tool to predict the clinical outcomes of ICB. Of note, scRNA-seq of CD8+ TILs correlates with the immunohistochemical (IHC) staining of tumor samples and thus reflects CD8+ infiltration in tumors [53]. The measurement of clonal expansion by single-cell TCR sequencing (scTCR-seq) revealed the patient’s unique repertoire and indicates that T cell clones reside not only within the tumor but also in the surrounding tissue and blood circulation [110]. Next-generation sequencing of TCR in CD8+ cells from biopsies of metastatic melanoma prior to treatment showed that a less diverse repertoire and thus a higher abundance of specific clones is accompanied by a better outcome of PD1 blockade therapy [49]. This may be due to the clonal expansion of melanoma-associated antigen -specific T cells at the side of the tumor [49,110]. Similar results have been reported in NSCLC [111]. However, Puig-Saus et al. (2023) observed fewer antigen-specific TCR clonotypes in individuals not responding to anti-PD1 therapy [112]. Such discrepancies could be attributed to clonal revival [53] and clonal replacement [113], in which T cell clones specific for a given epitope emerge successively and the existing clones diminish and re-emerge over time. Interestingly, blocking both PD-1 and LAG-3 resulted in a high enrichment of new clones in the TME with a terminally exhausted and activated phenotype in patients with advanced melanoma and HNSCC. [54,59]. The synergistic effect of this combination therapy directly impacts signals 1 and 2 in the T cells, since LAG-3 inhibits TCR signaling, whereas PD1 primarily inhibits co-stimulatory signals provided by CD28 [64].

### 4.3. PET Imaging of CD8+ T Cells Biodistribution

Positron emission tomography (PET) scans provide noninvasive whole-body imaging data for tracking CD8+ T cells in patients with solid tumors undergoing immune checkpoint blockade. The tracers have been proven safe, with no side effects [101]. These authors quantitatively visualized CD8+ cells labeled with zirconium-89 (89Zi) and followed their dynamics in cancer patients before and after the administration of PD(L)1 and/or CTLA-4 inhibitors. Of note, metastatic lymph nodes and inflamed lesions had a significantly higher standard uptake value (SUVmax) compared to healthy lymph nodes and healthy tissue, and patients with a higher SUVmax had a better OS, likely due to a greater amount of CD8+ T cells activated in secondary lymphoid organs (e.g., spleen, lymph nodes) and subsequently in infiltrating tumor lesions. In line with these observations, 89Zi uptake in the tumor was higher in patients with mismatch repair deficiency (dMMR) than in MMR-proficient (pMMR) patients, which is consistent with reports of enhanced CD8+ T cell infiltration in dMMR tumors.

It is worth mentioning that CD8 is not only expressed on the surface of T cells but can also be present on NKs and DCs. Thereafter, radioactive tracer antibodies targeting inhibitory receptors have been developed, including 89Zi-labeled anti-PD1 [102,103] and its ligand anti-PD-L1 [114]. Both tracers showed that tumor uptake predicts therapeutic response in patients undergoing ICB [102,103,114]. However, 89Zi-labeled anti-PD1 did not distinguish between PD1-expressing T cell subsets (e.g., CD8 vs. CD4, cytotoxic vs. immunoregulatory) or other immune cells like NK, B cells, macrophages, and dendritic cells that might express this molecule [115], and no correlation was found between CD8 expression and the tracer [102].

This approach could be employed not only to track T cells in response to ICB but also as a predictive biomarker to characterize tumor infiltration by treatment-naïve CD8+ cells. Furthermore, it can also identify the potential development of T cell-associated irAEs during treatment by detecting an increased infiltration of CD8+ T cells in non-malignant tissue. For instance, Kist de Ruijter et al. (2022) observed PET changes in a patient developing Hashimoto’s thyroiditis as a consequence of ICB treatment [101].

The limitations of this approach lie in the biased interpretation of the imaging of radiolabeled CD8+ T cells, in changes in cell biodistribution before, during, and after treatment, and in the large heterogeneity in CD8+ cell distribution, not only between patients but also within an individual [101].

### 4.4. Three-Dimensional Co-Culture Systems: Advancing TME Modeling and Cancer–Immune Crosstalk

Three-dimensional (3D) co-culture systems have emerged as powerful tools for simulating the in vivo tumor microenvironment and studying cancer–immune interactions with greater physiological relevance than traditional 2D cultures. These systems—primarily spheroids and organoids—better recapitulate key aspects of the TME, including extracellular matrix composition, spatial organization, and dynamic cell–cell interactions [116]. Spheroids, which are 3D cell aggregates typically formed from a single cell type, can be generated without scaffolds using hanging drop, suspension cultures, or 3D bioprinting methods [117]. Importantly, larger spheroids can develop oxygen and nutrient gradients, displaying gene and protein profiles more resembling in vivo conditions [116,118]. By integrating cancer cells with various immune components, such as TILs, MDSCs, and macrophages, 3D co-culture platforms enable detailed investigations into immune evasion, checkpoint regulation, and therapeutic responses [116,119]. Their relevance and potential for studying T cell behavior was recently demonstrated by Ou and colleagues (2022), who used patient melanoma-derived spheroids to assess T cell infiltration and activation, incorporating CAFs to observe their suppressive effects [119]. Moreover, spheroids are particularly useful for high-throughput screening, e.g., identifying factors that influence T cell exhaustion, although this remains largely unexplored.

In this context, we developed an in vitro testing system to predict the therapeutic efficacy of anti-PD1 therapy in patients prior to initial ICB treatment. Using a melanoma-CSC spheroid co-culture system with CD8+ T cells from healthy donors, we induced an exhausted T cell phenotype (“co-culture of exhaustion”) and assessed the exhaustion profile and reactivation potential of effector CD8+ T cells following anti-PD1 treatment. The preliminary data suggest that CSCs upregulate key exhaustion markers, including PD-L1 and CTLA-4. By evaluating T cell activation, expansion, and exhaustion markers, we found initial evidence that CD8+ T cells become exhausted in the presence of melanoma CSC spheroids, consistent with the immune evasion characteristics associated with CSCs. As a proof of principle, we are extending this system to blood samples from patients with metastatic melanoma, which allows us to monitor and predict the efficacy of ICB treatment in these individuals. The application of scRNA-seq and cytokine assays provides deeper insight into the regulation of molecular feedback pathways. Together with patient-derived data, these could be used in the artificial intelligence (AI)-assisted computational modeling of the complex cancer–immune cell crosstalk to support clinical decision making in immunotherapy. Here, recently developed systems such as LORIS or SCORPIO have already shown impressive performance and accuracy. In particular, the latter system, which uses routine blood tests and clinical data, outperformed two currently FDA-approved biomarkers, TMB and PD-L1 immunohistochemistry, in predicting responses to checkpoint inhibitors. However, the development of machine learning models is still ongoing, as more research is needed to prospectively validate the use of these models in different clinical settings [120,121,122].

Given the role that CSCs play in cancer progression, they also represent intriguing targets for adenoviral vector (AdV)-mediated gene therapy. By coupling peptides or aptamers specific to CSC surface proteins such as CD44 or CD133 with AdVs, we can selectively target and reprogram these highly malignant cells to either block ICB production or create an immunoinvasive environment. Such an approach was successfully demonstrated by Ascic and colleagues (2024). They utilized human cancer spheroids co-cultured with immunosuppressive CAFs, MDSCs, or pericytes to study immune reprogramming. By combining this system with adenoviral gene delivery, they were able to convert certain cancer cells into conventional DC type 1, which promoted T cell activation and the elimination of tumor cells. Translation of this approach to immunocompetent mouse models demonstrated its efficacy in reducing exhausted and regulatory T cell populations, while enhancing the recruitment and expansion of polyclonal cytotoxic and memory T cells. Importantly, they also found synergistic effects with anti-PD1 and anti-CTLA-4 applications compared to monotherapy [123].

Despite their advantages, challenges remain such as standardization, scalability, and the inclusion of vascular and stromal elements to fully mimic in vivo conditions. Nonetheless, these advanced models incorporating patient-derived cells or organoid technologies represent a crucial step toward improving preclinical cancer models by increasing translational potential and enabling personalized immunotherapy strategies.

## 5. Conclusions and Future Perspectives

Overcoming exhaustion and reactivating tumor-killing CD8+ T cells by blocking inhibitory receptors has improved OS in cancer patients. ICBs as a monotherapy or combined agents (e.g., PD1 and LAG-3) have been tested in numerous clinical trials, with promising success in tumor reduction. However, low response rates and irAEs represent a major challenge that could be solved by stratifying responsive patients. Reliable biomarkers for ICB therapy in cancer cells remain controversial, with results differing between studies due to variability in study design, treatment regimens, patient cohorts, sampling techniques, and clinical characteristics. A major contributing factor is the diversity of CD8+ T cell subsets, which plays a central role in tumor regression after ICB therapy. Standardized criteria for T cell characterization, including uniform sampling techniques and biomarker consensus, are decisive to achieve consistent predictive outcomes. Modern imaging techniques such as PET scans provide insights into the distribution of CD8+ T cells, but patient tumor heterogeneity may limit reliability. In contrast, patients with higher concentrations of intratumoral and circulating Tc1, progenitor-exhausted, and Tem cells tend to benefit more from ICB therapy, highlighting the need to assess not only their abundance but also their functionality and reactivation potential. Peripheral CD8+ T cells as primary ICB targets offer a promising avenue for biomarker development when studied in standardized in vitro systems using patient material.

Boosting anti-tumor T cell function has been pursued by other means. CAR T cells and genetically TCR-engineered T cells that recognize TAAs or tumor-specific antigens have shown efficacy in hematological malignancies and are currently being tested in solid cancers [124,125]. T cell engagers (i.e., bispecific and trispecific antibodies and molecules) aim to increase tumor-specific targeting and are tested in ongoing clinical trials for both liquid and solid cancers. These engagers bind tumor antigens presented on MHC-I on one side (e.g., HLA.A2:gp100), and on the other side they bind to CD3/CD28/CD137 presented on T cells. Furthermore, the primary focus of these new approaches is to increase safety and efficacy. T cell engagers with peptide-covered antibody-binding regions that are only available after processing by TME-specific proteases are under current investigation [16].

Eventually, the use of targeted gene therapeutic tools such as adenoviral vectors, adeno-associated viruses, or lipid nanoparticles to reprogram and manipulate cancer cells and their TME may help to reduce the immunosuppressive environment. The ongoing discovery of novel antigens through experimental and bioinformatics approaches will provide new target structures for these systems and ICBs. Thus, combination therapies targeting both the TME and the immune system are emerging as promising approaches [16,126].

Finally, computational modeling and bioinformatics approaches can improve biomarker discovery and predictive accuracy. The integration of CD8+ T cell signatures—assessing exhaustion markers, immune infiltrates, functionality, TCR diversity, and effector–regulatory balance—together with other tumor-intrinsic immunological and genetic markers will refine patient stratification and personalization of therapies.

## Abbreviations

The following abbreviations are used in this manuscript:

AdV	Adenoviral vector
CAF	Cancer-associated fibroblast
CSC	Cancer stem cell
CyTOF	Cytometry by time of flight
DC	Dendritic cell
FC	Flow cytometry
FFPE	Formalin-fixed paraffin-embedded
GC	Gastric cancer
HNSCC	Head and neck squamous cell carcinoma
IHC	Immunohistochemistry
irAE	Immune-related adverse events
MDSCs	Myeloid-derived suppressor cells
NK	Natural killer cell
NSCLC	Non-small cell lung cancer
PB	Peripheral blood
PBMCs	peripheral blood mononuclear cells
PET	Positron emission tomography
scRNA-seq	Single cell RNA sequencing
SKCM	Skin cutaneous melanoma
TCR	T cell receptor
TILs	Tumor infiltrating lymphocytes
TME	Tumor microenvironment
TNBC	Triple-negative breast cancer
